# Factors Influencing Stress in Families of Individuals with Illness or Disability: A Qualitative Study Based on Family System Unit Stress Theory

**DOI:** 10.3390/healthcare14081081

**Published:** 2026-04-18

**Authors:** Aoba Kadono, Naohiro Hohashi

**Affiliations:** Division of Family Health Care Nursing, Department of Nursing, Graduate School of Health Sciences, Kobe University, Kobe 654-0142, Japan; 250k003k@stu.kobe-u.ac.jp

**Keywords:** family stress, influencing factor, family nursing, family system unit stress theory

## Abstract

**Highlights:**

**What are the main findings?**

- Qualitative interview surveys indicated factors influencing stress occurring in families with young/adult children with illnesses or disabilities.
- Using Family System Unit Stress Theory as the theoretical framework, both positive and negative factors influencing family stress were systematically classified.

**What are the implications of the main findings?**

- Factors influencing family stress occurring in families with young/adult children with illnesses or disabilities accumulate as the family grows and develops, transforming family stress over time.
- To promote effective adaptation to family stress, an integrated approach to influencing factors should be taken, reducing risk/causal/promoting factors and strengthening preventive/inhibitory/suppressive factors.

**Abstract:**

**Background/Objectives:** Stress experienced by families of individuals with illnesses or disabilities is shaped through the interaction of multiple complex influencing factors. This study aimed to elucidate the factors influencing stress in families of young/adult children with illnesses or disabilities, using Family System Unit Stress Theory (FSUST) as the guiding theoretical framework. **Methods:** Semi-structured interviews were conducted with 10 families of young/adult children with illnesses or disabilities. Data were analyzed using qualitative content analysis following the approach of Graneheim and Lundman. In line with FSUST, the identified influencing factors were categorized into negative factors (risk/causal/promoting) and positive factors (preventive/inhibitory/suppressive). **Results:** A total of six categories and 18 subcategories were extracted for the risk/causal/promoting factors, including “accumulation of unshared burdens within the family leading to role overload” and “concerns about the future of the young/adult child.” For the preventive/inhibitory/suppressive factors, five categories and 13 subcategories were extracted, including “receiving a diagnosis of the young/adult child’s illness or disability” and “family maintaining a positive attitude.” **Conclusions:** Family stress in families of young/adult children with illnesses or disabilities varies through the interaction of multilayered influencing factors, including persistent emotions carried over from the past, difficulties faced in the present, and anticipatory concerns regarding the future. Therefore, nursing practice requires a life course-oriented understanding of family stress and an integrated approach that concurrently reduces risk/causal/promoting factors while enhancing preventive/inhibitory/suppressive factors.

## 1. Introduction

Family stress emerges as a response to the numerous life events encountered by families, such as illness or disability of a family member, births and deaths, and changes in living environments [1]. Family stress is shaped not only by major stressors that directly provoke stress but also by the accumulation of minor stressors arising in daily life. In addition, factors internal to the family, influences external to the family system, and the family’s appraisal of stress may collectively contribute to the escalation of family stress [2].

Factors influencing family stress are not limited to those with negative effects. Family stress is shaped by both negative and positive factors, and family adjustment and adaptation occur through the dynamic interplay between these opposing influences [1]. For example, family resilience has recently gained increasing attention as a positive influencing factor that enables families to not only overcome crises but also experience growth in the face of adversity [3]. In addition, factors such as hope, optimism, and positive coping have been identified as influences that mitigate family stress [4]. When providing family support, health care professionals should seek to not only remove or attenuate factors that negatively affect the family but also promote positive coping behaviors within the family and enhance the availability of resources and social support that facilitate such adaptive responses [5].

Therefore, clarifying both negative and positive influencing factors may enable the development of effective interventions for family stress. However, compared with the broader body of research on stress, research focusing on factors influencing family stress remains limited, and important gaps persist [6,7]. Moreover, the interaction between negative and positive factors that influence stress at the level of the family as a whole has not been sufficiently elucidated. The complex factors influencing family stress and the ways in which they operate merit qualitative research that captures families’ narratives in depth.

Families that include a member with illness or disability are exposed to numerous stressors in daily life and are more likely to experience elevated levels of family stress [8]. According to Soh et al. [9], approximately one in three caregivers experience depression and/or anxiety, and almost one in two caregivers experience caregiver burden. This burden can be dispersed as stress to the entire family. For example, children with special care needs often require greater parental attention and care than typically developing children, which can function as a stressor and may lead to high levels of family stress and declines in family functioning [10]. In addition, for families of young/adult children with illnesses or disabilities, effectively managing daily conflicts and stress is essential to cope with the diverse challenges encountered in everyday life [11]. Furthermore, the stress process in these families is rendered more complex by the operation of multilayered influencing factors both within and outside the family system [12,13]. Accordingly, professionals need to identify the dynamic stressors affecting families and provide support for family stress management [14]. Clarifying the factors that influence stress in families that include a member with illness or disability is expected to contribute to more effective family support aimed at enhancing family well-being.

In recent years, interest in families that include a member with an illness or a disability has increased [15]. While much research has been conducted on caregiver burden, parenting stress, and family functioning, most of this research has focused on specific family members—such as mothers or primary caregivers—while paying limited attention to the family as a whole. Moreover, within the literature on illness and disability, relatively few studies have explicitly examined stress at the level of the entire family system [16]. Moreover, much of the research to date has examined individual influencing factors in isolation, and the negative/positive influencing factors and their combined effects are not well understood. Although a substantial body of research has addressed families with children in the childrearing stage, comparatively little attention has been paid to families composed of adult children and aging parents. Indeed, perspectives examining stress across diverse family life stages remain underdeveloped [15]. These gaps in knowledge highlight the need for research that dynamically and comprehensively captures family stress throughout the entire life course of families that include a person with illness or disability.

These challenges can be addressed by a theoretical framework that conceptualizes stress at the level of the entire family as a dynamic process. Hohashi [1] proposed the Family System Unit Stress Theory (FSUST) to explain the emergence of family stress when stressors act upon the family and precipitate a family crisis, as well as the processes of family adjustment or adaptation and subsequent recovery and regeneration. By capturing the dynamic process of stress at the level of the family system over time, FSUST helps visualize how families overcome adversity. By conceptualizing the family as both a system and a unit, FSUST enables clarification of the complex stress processes experienced by the family as a whole. Furthermore, FSUST provides a structured approach for systematically analyzing complex family stress by clearly distinguishing among family stressors, family stress itself, and the factors influencing family stress within the stress process. The present study aimed to clarify the factors influencing stress among families of young/adult children with illnesses or disabilities, using FSUST as the theoretical framework. The overarching research questions are as follows: (1) What negative/positive factors influence family stress in families of young/adult children with illnesses or disabilities? (2) How do these negative/positive influencing factors work in shaping the family stress process? Addressing these issues seeks to contribute to the development of more effective intervention strategies for family stress at the family system unit level.

## 2. Methods

### 2.1. Operational Definition of Terms

- The term “family system unit” is used as an alternative expression for “family” to emphasize that the family is conceptualized simultaneously as a system and as a unit [1].
- “Risk/causal/promoting factors” are defined as factors that predispose the family system unit to the emergence of stress, precipitate the onset of family system unit stress, or intensify the severity of stress once it has emerged [1].
- “Preventive/inhibitory/suppressive factors” are defined as factors that protect the family system unit from the emergence of stress, impede the development of family system unit stress, or attenuate the severity of stress once it has occurred [1].

### 2.2. Study Design

This study employed a qualitative descriptive design to explore in depth the experiences related to family system unit stress. A qualitative descriptive design is appropriate for providing a straightforward description of a phenomenon and for gaining insight into participants’ perceptions and experiences [17]. The study conducted semi-structured interviews for data collection and used qualitative content analysis to analyze the data. This method allows for the maintenance of trustworthiness in nursing research while enabling interpretation that encompasses both the obvious components of the data and their underlying meaning [18].

### 2.3. Participants

Participants in this study were selected using purposive sampling and recruited using convenience sampling. This sampling approach was adopted with the aim of deeply exploring and describing the stress experiences of young/adult children with illness or disability while ethically and practically ensuring the feasibility of access to their families [19]. Eligibility criteria included being families living with a family member who had an illness or disability, willing to participate in the study, and able to sign a consent form. The study placed no restrictions on the type or severity of the illness or disability, nor on the age of the affected family member. Exclusion criteria included inability to participate in interviews in Japanese. We recruited participants from two child development support centers, one after-school day service center, one community activity support center, and one life care service center in Japan. With the cooperation of these facilities, the researcher distributed invitation letters describing the study to 203 service users. Families who expressed interest in participating contacted the researcher directly to discuss the date and method of the interview. They were provided with a detailed explanation of the study’s purpose and procedures, as well as assurances regarding the protection of personal information and anonymity. Participant recruitment was conducted between 2024 and 2025.

### 2.4. Theoretical Framework

**Figure 1:**
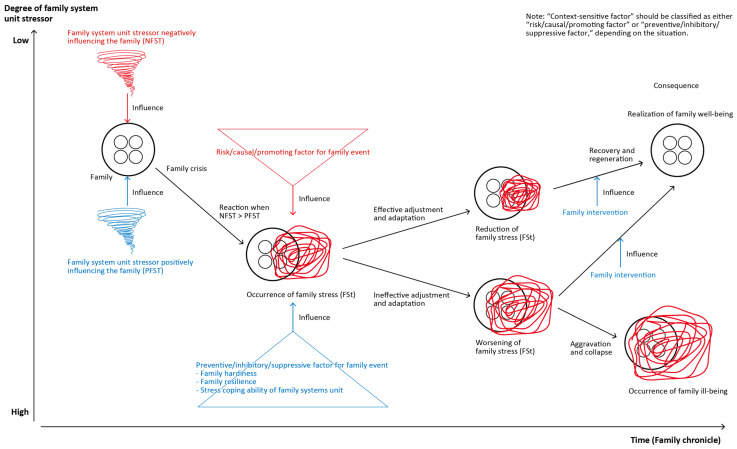
Model diagram of Family System Unit Stress Theory (FSUST; ver. 2.2).

This study adopted FSUST, proposed by Hohashi [1], as its theoretical framework. FSUST is a family nursing practice theory that focuses on the relationship between family system unit stressors and family system unit stress. The theory presents, in chronological sequence, the process by which a family is exposed to family system unit stressors, develops family system unit stress, and subsequently undergoes recovery and regeneration (Figure 1). In FSUST, a family system unit stressor refers to an event occurring within the family system unit that functions as a stimulus producing family system unit stress. Family system unit stress refers to the family symptoms or signs that arise within the family system unit in response to these stressors.

Positive and negative family system unit stressors operate within a family. When negative family system unit stressors outweigh positive ones, family system unit stress emerges. Family system unit stress is further influenced by various factors, which are classified as risk/causal/promoting factors and preventive/inhibitory/suppressive factors. Although these factors function antagonistically, when the latter outweigh the former, effective family adjustment and adaptation occur, leading to the realization of family well-being. As such, FSUST enables the process by which influencing factors act on family system unit stress to be understood within the unique context of each family. Moreover, applying FSUST to analyze the phenomenon enables researchers to distinguish and systematically organize the complex risk/causal/promoting factors and preventive/inhibitory/suppressive factors operating within families. For these reasons, we considered FSUST an appropriate theoretical framework for the present study.

### 2.5. Procedure

Interviews were conducted either face-to-face or via an online video conferencing system, according to participants’ preferences. Regardless of the format, interviews were held in a private room to ensure confidentiality, and only the participant and the researcher were present. Each interview was conducted in Japanese and lasted approximately two hours. The interviews were audio-recorded with the participants’ consent.

To obtain data related to the research objectives, an interview guide including open-ended questions was created. The interview guide was developed based on FSUST and used to encourage participants to describe their experiences in their own words. In semi-structured interviews, the order and content of questions were flexibly adjusted to meet the participants’ narratives. The interview guide included the following questions: “From your perspective, what people, situations, or events have influenced the difficulties or stress experienced by your family as a whole?” “In what ways, if any, did those people, situations, or events affect your family as a whole?” “In what ways did those people, situations, or events intensify or alleviate stress within your family as a whole?” “Reflecting on the past, what people, circumstances, or events do you think influenced the difficulties or stress experienced by your family as a whole?” “In the future, what people, circumstances, or events do you anticipate might affect the difficulties or stress experienced by your family as a whole?” In this way, participants were asked about stress levels within their entire family, not individual stress levels.

### 2.6. Data Analysis and Rigor

This study used Graneheim and Lundman’s approach to qualitative content analysis [18]. The data collection and analysis were conducted concurrently. Immediately after each interview, the audio-recorded data were transcribed verbatim in their entirety. The transcripts were read repeatedly by the primary researcher and a co-author to achieve an accurate and comprehensive understanding of the overall content. Next, the study extracted descriptions related to factors influencing stress in the family as a whole, which were then condensed into individual codes. During coding, we took the broader context of the text into account and continuously compared each code with others based on similarities and differences. In the subsequent step, we grouped codes into subcategories and categories according to shared meanings. The categories were discussed and refined through consensus among a third party consisting of 12 researchers with expertise in family nursing. Finally, based on FSUST, the study classified the categories into risk/causal/promoting factors and preventive/inhibitory/suppressive factors.

In this study, methodological rigor was ensured in accordance with Lincoln & Guba’s rigor criteria [20]. Credibility was enhanced through the collection and analysis of detailed interview data. Dependability and confirmability were supported through a transparent and systematic qualitative content analysis process based on the theoretical framework of FSUST. Transferability was facilitated by providing detailed descriptions of participant selection, data collection methods, and analysis processes.

The analysis in this study was conducted by researchers who are nurses interested in family stress and family support. Therefore, recognizing the possibility that the researchers’ own professional backgrounds and values might influence data interpretation, the analysis proceeded with a reflective attitude, faithfully returning to the content of the verbatim transcripts. Furthermore, the context of the participants’ narratives was respected, and efforts were made to avoid excessive interpretation due to the researchers’ preconceptions. In addition, the theoretical framework used in this study was developed by a co-author who was aware of the influence of his own research background and positionality as a theorist on the analysis, and who accordingly made efforts to mitigate bias through discussions and comparisons of data interpretation within the team.

Data collection continued until saturation was achieved. Saturation at the level of subcategories was reached after interviews with nine families [21]. Two researchers evaluated whether data saturation had been attained; to ensure the robustness of data saturation, the study interviewed one additional family. In total, the analysis included data from 10 families.

### 2.7. Ethical Considerations

This study was conducted after review and approval by the institutional review board of the university with which the researchers are affiliated. All participants took part voluntarily and were provided, both in writing and verbally, with explanations regarding the purpose and procedures of the study, voluntary participation, the right to withdraw or discontinue participation at any time, anonymity, audio recording of interviews, and publication of the findings. Written informed consent was obtained from all participants prior to data collection. In reporting the results, the researchers anonymized all information that could potentially identify individuals to protect participants’ privacy.

## 3. Results

### 3.1. Sociodemographic Characteristics of the Participants and Their Young/Adult Children

The characteristics of the 10 families are presented in Table 1. The interview participants consisted of 10 individuals aged 31 to 75 years (nine females and one male). All families had a nuclear family structure. The characteristics of the 12 young/adult children are presented in Table 2. The young/adult children with illnesses or disabilities included 12 individuals aged 5 to 40 years (eight females and four males). Regarding siblings of these young/adult children, there were no siblings in one family, one sibling in six families, and two siblings in three families.

### 3.2. Categories and Subcategories of Risk/Causal/Promoting Factors

Through content analysis, the study identified six categories and 18 subcategories of risk/causal/promoting factors (Table 3). Examples from the families’ narratives are presented in italics. All quotes from the interviews have been translated and edited for readability.

#### 3.2.1. Presence of Symptoms or Condition-Related Characteristics in the Young/Adult Child

Young/adult children exhibited characteristics such as impulsive behaviors, tantrums, and aggressive behaviors toward others, and often engaged in unpredictable actions. As families confronted these characteristics on a daily basis, they frequently experienced parenting as difficult and, at times, lost confidence in their caregiving abilities. In addition, young/adult children with mental illness at times experienced worsening depression or psychosis, which made daily life increasingly challenging. Continually attending to a psychologically unstable young/adult child led to emotional exhaustion for the families. Irritation arising in this context often extended to siblings, creating additional psychological burden. Some families also reported feeling guilty toward siblings because support for the young/adult child was consistently prioritized.

*She would cry intensely, run around and try to escape, and even when we went to the park, she would often try to leave almost immediately. I was constantly in a state of confusion, worrying that she might run into the road.*

(ID 7)

#### 3.2.2. Difficulties in the Young/Adult Child’s Interactions with Others

Some young/adult children were unable to speak or had difficulty engaging in conversation, which prevented effective communication of their intentions to others. In addition, misunderstandings frequently occurred in their communication, making it difficult for them to establish and maintain long-term interpersonal relationships. Families expressed significant concern that these characteristics could lead to social isolation. The parents also reported that their young/adult children often did not accept intervention from others, which limited access to social support. Even when the parents attempted to obtain support, the young/adult child’s refusal created situations that generated family conflict and feelings of helplessness.

*My son absolutely refuses to leave the house and doesn’t want to go to the support center. Once he decides he doesn’t want to go, he won’t listen to anything I say, and I can’t force him to go.*

(ID 10)

#### 3.2.3. Difficulties in Parenting and Supporting a Young/Adult Child with Illness or Disability

Before the illness or disability had been identified, the parents described experiencing a sense of discomfort or doubt regarding their young/adult child’s perceived lack of adaptability. The parents struggled with how to respond, often attempting to cope by providing strict guidance or encouragement. Even after the diagnosis was established, the parents reported continued difficulty in understanding the young/adult child’s thoughts and feelings. Discrepancies in perspectives led the parents to experience frustration and a sense of helplessness. The parents also expressed internal conflict about not being able to adequately empathize with or support the difficulties and distress faced by their young/adult child. Moreover, even as the young/adult child grew older, the parents described ongoing regret about how they had responded in the past. These challenges in parenting and support contributed to the accumulation of frustration in both parents and young/adult children, thereby intensifying family stress.

*I feel that by trying to force my son into what is considered “normal,” I have gradually taken away the happy, lively side of him that was truly his. That inner conflict has become a major source of stress for me.*

(ID 5)

#### 3.2.4. Accumulation of Unshared Burdens Within the Family Leading to Role Overload

The parents described feelings of guilt regarding the fact that their young/adult child was born with an illness or disability and expressed a belief that they themselves had to cope with the problems faced by their child and family. They also reported that the more serious the family’s difficulties or concerns became, the less able they felt to consult others. Even when they did seek support, their distress was often not fully understood. Casual remarks intended to provide comfort or advice that missed the point led families to feel that their struggles were not truly recognized, eventually discouraging them from relying on others. Role overload was particularly evident among mothers. The mothers reported that while fathers were at work, they alone assumed responsibility for caregiving and support. In some cases, the fathers did not attempt to understand the young/adult child’s symptoms or characteristics and were not actively involved in caregiving or support. Such circumstances limited the mothers’ opportunities for social interaction and intensified emotional strain and feelings of isolation, thereby amplifying stress within the family as a whole.

*My husband would leave early in the morning and not come home until around 10 p.m. I was the one bathing our daughters, feeding them, and putting them to bed—I was raising them on my own the entire time. I felt cut off from society, as if I were being left behind, and that was very painful.*

(ID 9)

#### 3.2.5. Insufficient Support for the Family

The parents reported that the absence of relatives on whom they could rely for assistance intensified their sense of difficulty in caregiving and support. In addition, limited options for services and future pathways for the young/adult child constituted a significant challenge for families. When the young/adult child’s disability was relatively mild, appropriate support was at times even more difficult to access. Some families living in rural areas also faced restricted availability of services and educational or vocational pathways. Furthermore, the parents stated that information regarding the illness and available support was insufficient. Vague explanations from health care professionals and limited support from government agencies contributed to feelings of dissatisfaction among families.

*We have no way of knowing what kinds of support are actually available to us, yet the local government and administrative agencies do not provide any information. They should be aware of individuals like my daughter who have illnesses or disabilities, but there is virtually no outreach or information provided to families like ours.*

(ID 8)

#### 3.2.6. Concerns About the Future of the Young/Adult Child

The parents expressed anxiety about the young/adult child’s life after the parents’ death. Families worried about the young/adult child’s future and sought better living arrangements and external resources to ensure the child’s long-term stability. At the same time, families stated that they hoped the young/adult child’s siblings would be able to live their own lives and did not want to place caregiving responsibilities on them in the future. These conflicting feelings generated internal tension within the family and, in certain cases, hindered open discussions about the family’s future.

*My daughter is kind, so she would never say that taking care of her younger sister is a burden. But she is married, and I feel that I shouldn’t impose on her. She lives far away, and in a way, I think it’s better that we can’t easily rely on her.*

(ID 4)

### 3.3. Categories and Subcategories of Preventive/Inhibitory/Suppressive Factors

The content analysis identified five categories and 13 subcategories of preventive/inhibitory/suppressive factors (Table 4). Examples from the families’ narratives are presented in italics.

#### 3.3.1. Receiving a Diagnosis of the Young/Adult Child’s Illness or Disability

Families reported that they had attended hospitals over an extended period to clarify the cause of the symptoms experienced by their young/adult children. Receiving an accurate diagnosis enabled families to understand the etiology of the symptoms, which alleviated their anxiety. The parents also stated that, prior to diagnosis, they had significant doubts and confusion regarding the perceived difficulty of raising their young/adult child. A formal diagnosis of illness or disability provided a sense of understanding and acceptance for the parents and contributed to the reduction in stress within the family as a whole.

*I couldn’t easily accept that my daughter had an illness, but at the same time, I felt relieved to finally understand the cause of her difficulties. During the year and a half after we began going to the hospital, I was constantly anxious, but I gradually came to understand that her struggles were caused by the illness.*

(ID 8)

#### 3.3.2. Parental Engagement with the Young/Adult Child Based on an Understanding of Their Characteristics

The parents described adapting their interactions to align with the characteristics of their young/adult children. In the course of caregiving and support, the parents made deliberate efforts to understand their children and actively modified the ways in which they communicated with them. The parents also reported that they provided caregiving and support with an awareness of the young/adult child’s future independence. Specifically, they offered careful guidance on daily living habits, sexuality-related issues, and appropriate ways of interacting with others so that their children could live safely and independently. These efforts helped reduce the young/adult child’s daily life burdens and had a positive impact on the family as a whole.

*Ever since she underwent developmental testing, I’ve strongly felt that I can’t support her forever. No matter what path she chooses, I believe she needs to become independent and be able to take care of at least some aspects of her daily life on her own, and that’s the mindset I have when I interact with her.*

(ID 6)

#### 3.3.3. Family Maintaining a Positive Attitude

Families coped with difficulties by accepting differing perspectives within the family and advice from supporters and by consciously reframing situations in a positive light. The parents reported that prioritizing the young/adult child’s happiness, believing that it was sufficient if the child could live contentedly, helped them overcome challenges. Families also actively engaged with the young/adult child’s supporters, teachers, and school peers, building long-term relationships. Furthermore, when the fathers understood the young/adult child’s illness or disability and actively participated in caregiving and support, the mothers experienced greater emotional and time-related flexibility, which had a positive impact on the family as a whole.

*I often think about how to help others see the fun and unique sides of my daughter. I want everyone to enjoy watching her grow together, so I make a point of telling her teachers about all of her good qualities.*

(ID 9)

#### 3.3.4. Adequate Support for the Family

The findings indicated that relatives’ close involvement in caregiving and provision of support for the young/adult child functioned as a significant source of support for the family. In addition, proactive engagement from supporters, such as speaking to the young/adult child and assisting with daily care, contributed to a reduction in family stress. Receiving such support enabled young/adult children to expand their abilities and improve their emotional self-regulation, which in turn alleviated family anxiety.

*Through receiving support, my daughter has become better able to put her feelings into words. In the past, she could only say things like, “I’m just irritated,” but recently, she has gradually begun to think through, step by step, how she can deal with those feelings.*

(ID 6)

#### 3.3.5. Availability of an Environment in Which the Family Can Consult Others

The parents reported that they coped with difficulties by actively consulting supporters regarding caregiving and support for their young/adult children. Having access to professionals with whom they could discuss problems arising in daily life contributed to a reduction in family stress. In addition, interaction with other families raising children with illnesses or disabilities helped alleviate the parents’ psychological burden. The parents stated that participating in parent associations and study groups for families of children with illnesses or disabilities, engaging in consultation and information exchange, and listening to others’ experiences contributed to improved caregiving and support within their own families.

*Even the worries that no one had understood before were accepted when I went to the mothers’ group. There, I found people I could talk to and rely on. Since joining the group, I’ve been able to approach parenting with much more emotional breathing room.*

(ID 5)

## 4. Discussion

### 4.1. Overall Picture

This study demonstrated that family stress experienced by families accumulates over time and constitutes a dynamic process shaped by the interaction of multilayered influencing factors at the individual, family, and societal levels. These findings are supported by the perspective that the family is both a system and a unit that continuously transacts with its internal and external environments [22]. By applying FSUST, the present study clarified the two antagonistic dimensions of influencing factors acting on family stress, namely, risk/causal/promoting factors and preventive/inhibitory/suppressive factors. These factors are believed to have functioned in a manner that either intensified or attenuated family stress over time. Furthermore, conceptualizing family stress along a temporal axis might indicate that not only past and present experiences but also anticipated future events serve as significant influencing factors.

### 4.2. Risk/Causal/Promoting Factors

The participating families described persistent feelings of guilt regarding the young/adult child’s illness or disability, beginning in early childhood and continuing into adulthood. Previous research has focused on families during the childrearing stage [23]; the present study is considered to indicate that parental guilt influences family stress across the entire life course.

The participating families also described how the characteristics and symptoms of their young/adult children made daily life unpredictable. The parents struggled to understand their children’s thoughts and feelings. Such parental psychological distress and diminished parenting self-efficacy may hinder effective caregiving and support, potentially escalating difficulties over time [24]. Even years later, parents continued to reflect on and question how they had responded to their young/adult children. Parents of young/adult children with illnesses or disabilities often continue to provide substantial support well into adulthood, and the chronic burden may accumulate over decades [25]. The present findings suggest that perceived caregiving difficulties and reduced self-efficacy are not transient experiences but may continue to influence family stress across the life course.

Anticipatory anxiety regarding future family events functioned as a significant influencing factor in family stress. The parents experienced chronic uncertainty about a future in which they would no longer be able to fulfill their caregiving roles [26]. Concerns about the young/adult child’s social participation and independence, as well as the potential caregiving burden placed on siblings, also influenced family stress [27]. Sustained anticipation of future stressful events may negatively affect emotions and cognition [28]. The present study is believed to indicate that such anticipatory anxiety extends beyond mere imagination about the future and operates as a factor that influences current family stress and caregiving or support behaviors.

The participants also experienced difficulty obtaining and utilizing information about available social support. One contributing factor was that when the young/adult child’s disability was relatively mild, they were more likely to fall outside the scope of existing systems and thus become caught in gaps between services, resulting in unmet support needs. The lack of accessible public information and concrete guidance regarding illness, disability, and available services further heightened family anxiety. Perceiving support from the state and society as inadequate has been shown to diminish overall family well-being [29]. The present findings indicate that broader social and structural factors surrounding the family may function as barriers to accessing support and services, thereby exacerbating family stress. After identifying such barriers, the authorities should provide holistic and systematic support aimed at promoting the social participation of individuals with disabilities and their families [30].

Role overload within the family also functioned as a barrier to the utilization of social support. The participating parents described a strong sense of duty and responsibility to manage their young/adult child’s needs on their own. Such beliefs may heighten feelings of guilt about relying on others and discourage the use of social support [31]. Moreover, the absence of fathers and limited access to external family resources contributed to a concentration of role overload among mothers. These circumstances may be influenced by cultural values prevalent in Asian contexts, including Japan, that discourage seeking support from others, as well as patriarchal norms that reinforce traditional caregiving roles [32,33]. Accordingly, social norms themselves that hinder collaborative caregiving and support within families need to be addressed.

### 4.3. Preventive/Inhibitory/Suppressive Factors

According to the participants, the diagnosis of illness or disability brought ambivalent emotions of grief and relief. The parents reported having felt discomfort or anxiety about their young/adult child’s behaviors even before a formal diagnosis was made [34]. The diagnosis provided an explanation for both the parents’ sense of incongruity regarding the young/adult child and the difficulties the family had experienced. Being able to understand these experiences generated a sense of relief. These findings suggest that diagnosis is not merely a matter of labeling but represents an important opportunity for families to assign meaning to previously uncertain experiences [35]. Continuous support from professionals before and after diagnosis, with attention to the family’s complex emotional responses, may promote effective adaptation and reduce family stress [36].

Conscious positive reappraisal also emerged as an important stress-coping ability for families. For example, the participants acknowledged their difficulties and anxieties while engaging in cognitive reframing and providing caregiving and support that prioritized the young/adult child’s well-being. During the adaptation process, the parents came to focus on what truly mattered in life and began to find new meaning within the context of disability [37]. These findings are believed to indicate that, across the life course, families gradually and intentionally reappraise their difficulties in a more positive light.

A well-established support environment for young/adult children reduced family anxiety related to caregiving and support and enhanced family capacity to cope with difficulties. According to Syed [38], the availability of social support significantly impacts the health and well-being of care providers. Furthermore, the provision of comprehensive social support is associated with reductions in family stress [13]. However, the mere availability of services does not necessarily lead to a decrease in psychological burden. Rather, family well-being is influenced by families’ recognition of available support and their ability to actively utilize it [39]. The present study highlighted the value of proactive engagement by supporters, such as the provision of information and practical guidance to families. For example, when proactive support was offered in response to family difficulties, families reported a heightened subjective sense of being supported. Furthermore, as families perceived the growth of their young/adult children, they reaffirmed the value of receiving support, which motivated the continued use of services.

In addition, interaction with peers who had undergone similar experiences was an important factor in reducing families’ psychological burden. By listening to peers’ experiences of caregiving and support, families were able to develop concrete expectations regarding caregiving and to prepare for the future. This suggests that anticipatory anxiety may be alleviated when families can foresee potential challenges based on experiential knowledge. The effectiveness of peer support for parents of children with disabilities has also been reported by Bray et al. [40], who recommended that professionals should actively inform families about such programs as an appropriate form of support.

### 4.4. Significance of Using FSUST as a Theoretical Framework and Its Implications for Family Nursing

A study using the ABCX model as a framework for family stress [41] noted that caregiving and support constitute an ongoing process and emphasized the need for research that examines influencing factors according to the child’s developmental stage. By employing FSUST as the theoretical framework, the present study could conceptualize the factors influencing family stress in families of young/adult children with illnesses or disabilities as cumulative across the child’s growth and development and as dynamically transforming family stress over time.

The present study revealed that family stress is shaped by the interplay of influencing factors, including emotions that persist from the past, challenges faced in the present, and anticipatory anxiety about the future. Furthermore, families were found to engage in meaning-making over time within their life course and to adapt effectively through various processes, such as positive reappraisal. These findings suggest that the emergence of family stress and processes of family adjustment and adaptation are dynamic and variable, influenced by the passage of time and changes in the support environment.

The current findings highlight the importance of understanding family stress across the life course and providing long-term support that anticipates future challenges. Therefore, family nursing practice requires an integrated approach that simultaneously removes or attenuates risk/causal/promoting factors while adding to or strengthening preventive/inhibitory/suppressive factors.

### 4.5. Limitations and Future Directions

This study has several limitations. Firstly, the representativeness of the sample is limited. While this study obtained diverse data by targeting families of young/adult children with various illnesses and disabilities, the small number of participants (*n* = 10) may limit the generalizability of the results. However, previous studies have suggested that data saturation in qualitative interviews in health research can be achieved with 9 to 17 participants [42], and several studies have been conducted with similar sample sizes [43,44]. Furthermore, it has been argued that data rigorously collected from small samples can adequately represent people’s experiences [45], suggesting that a small sample size does not necessarily impair the quality of the results. Moreover, although the study explored the influencing factors of stress occurring within family system units, a single-informant design was used, with many participants being the mothers of young/adult children. Nevertheless, it must be considered that the results may not fully reflect the context of stress in the family as a whole. Future research should strengthen the perspective of conceptualizing the family as a system unit and continue investigating with a larger number of participating families.

Secondly, limitations may exist due to the approach used in this study. This study is a cross-sectional study, and data on past influencing factors of family stress were obtained through participants’ reflections, while data on future influencing factors were obtained through participants’ predictions. Therefore, the possibility of recall bias and prediction bias must be considered. Furthermore, because participants were recruited through support facilities using convenience sampling, families not utilizing support services were not included in this study, resulting in the possibility of selection bias.

## 5. Conclusions

This study investigated the factors influencing family stress among families of young/adult children with illnesses or disabilities. The influencing factors of family stress were identified through qualitative analysis using semi-structured interviews grounded in FSUST. Family stress was not a one-time reaction to a specific event; rather, it was dynamically shaped by multilayered influencing factors at the individual, family, and societal levels. Furthermore, this study demonstrated that two types of influencing factors—risk/causal/promoting and preventive/inhibitory/suppressive factors—operate across past, present, and future temporal dimensions. By organizing family stress in both temporal and structural terms, the study contributes to strengthening the theoretical framework of FSUST.

## Figures and Tables

**Table 1:** Sociodemographic characteristics of the family members (*n* of families = 10).

ID	Participant							
	Age	Sex	Civil Status	Relationship to Young/Adult Children	Primary Caregiver	Family Size	Employment	Domicile
1	31	Female	Married	Mother	Yes	4	Homemaker	Rural
2	75	Female	Married	Mother	Yes	4	Retired	Urban
3	66	Female	Married	Mother	Yes	4	Retired	Urban
4	65	Female	Married	Mother	Yes	5	Retired	Urban
5	39	Female	Married	Mother	Yes	4	Self-employed	Urban
6	36	Female	Divorced	Mother	Yes	3	Employed full-time	Rural
7	38	Female	Married	Mother	Yes	4	Homemaker	Urban
8	53	Male	Married	Father	No	5	Employed full-time	Urban
9	40	Female	Married	Mother	Yes	5	Homemaker	Urban
10	52	Female	Married	Mother	Yes	3	Homemaker	Urban

**Table 2:** Sociodemographic characteristics of the young/adult children (*n* of families = 10).

ID	Young/Adult Children with Illnesses or Disabilities		
	Age	Sex	Main Diagnosis
1	6	Male	Developmental delay
2	32	Female	Intellectual disability
3	40 38	Female Male	Mild intellectual disability, mild paresis Mild intellectual disability, cerebral palsy, depression
4	30	Female	Schizophrenia
5	12	Male	Developmental disorder
6	9	Female	Developmental disorder
7	5	Female	Autism
8	12	Female	Rett syndrome
9	12 6	Female Female	Prader–Willi syndrome Autism
10	20	Male	Intellectual disability

**Table 3:** Categories and subcategories of risk/causal/promoting factors extracted from the content analysis.

Category	Subcategory	Code	Related Interviews (ID Number in Table 1)
Presence of symptoms or condition-related characteristics in the young/adult child	Feelings of parenting difficulty associated with the young/adult child’s characteristics	Feeling frustrated about the sudden actions of young/adult child Feeling of parenting difficulty caused by young/adult child’s tantrums Feeling drained by young/adult child repeating the same things over and over Worrying that young/adult child is not developing good habits	1, 5, 6, 7, 9, 10
	Family burden owing to symptom exacerbation in the young/adult child	Worrying about deterioration in young/adult child’s symptoms of depression The mental symptoms of young/adult child becoming a burden on the family Feeling fatigued from dealing with the positive symptoms of young/adult child	3, 4
	Sibling frustration related to the young/adult child’s symptoms or characteristics	Sibling becoming frustrated by the behavior of young/adult child Sibling feeling frustrated because his or her parents take out their frustrations on him or her after becoming exhausted from dealing with their young/adult child Sibling feeling unhappy that young/adult child always receives priority treatment	5, 6, 8
Difficulties in the young/adult child’s interactions with others	Difficulties in the young/adult child’s communication with others	Young/adult child having difficulty communicating with people outside the family Young/adult child not speaking and being unable to express his or her own thoughts	2, 5, 6, 8, 10
	Difficulty for the young/adult child in maintaining interpersonal relationships	Young/adult child’s inability to build relationships at support facilities Young/adult child’s relationships with support staff deteriorate and he or she stops going to the facility Young/adult child being unable to maintain friendships and becoming alienated Young/adult child has difficulty in building personal relationships and withdraws into his or her home	4, 5
	Refusal of support from others by the young/adult child	Young/adult child refuses to go to support facilities and stays at home Young/adult child shows resentment towards support from others Young/adult child refuses to be examined by a doctor	4, 10
Difficulties in parenting and supporting a young/adult child with illness or disability	Parenting and supporting the young/adult child amid uncertainty prior to diagnosis	Parents’ unawareness of the disability and worrying about the clumsiness of their young/adult child Parents’ unawareness of the disability and harshly scolding their young/adult child for his or her dysfunctional condition Families attempting to manage the symptoms of their young/adult child on their own before obtaining a diagnosis	1, 3, 4, 5, 6, 7
	Difficulty in understanding the young/adult child’s thoughts or perspectives	Not knowing what their young/adult child is worried about Difficulty in understanding the reasons behind their young/adult child’s thoughts and actions Parents concerned when their young/adult child says, “You don’t understand me”	3, 4, 7
	Parental conflict arising from difficulty empathizing with the young/adult child	Parents regretting not being able to support their young/adult child in overcoming his or her difficulties Parents struggling to force their young/adult child into a “normal” mold Parents struggling to adjust their family to the characteristics of their young/adult child	1, 3, 4, 5, 6, 7
Accumulation of unshared burdens within the family leading to role overload	Parental guilt regarding the young/adult child’s illness or disability	Parents feeling guilty about their young/adult child having disabilities Parents feeling responsible for matters concerning their young/adult child living on their own Parents feeling pressured over having a child with a disability	1, 3, 5, 8, 9
	Difficulty communicating parental concerns to others and receiving understanding	Parents finding it difficult to communicate their struggles to others Parents finding it difficult to get friends to understand the challenges they face in parenting and support The lack of knowledge and understanding of disabilities and support from those around them	2, 3, 4, 5, 6, 7, 9
	Insufficient father involvement in caregiving and support	Fathers being too busy with work to assist adequately with parenting and support Fathers failing to understand parenting and support for young/adult child Insufficient father participation, leaving mothers to shoulder the burden of childcare alone	2, 3, 4, 6, 7, 9, 10
Insufficient support for the family	Absence of relatives who can provide support	Relatives living far away and unable to assist with childcare Relatives who could be relied upon have moved far away, making it no longer possible to receive support	4, 7
	Limited options for support services and future pathways	Young/adult child has mild disabilities, resulting in limited availability of support Scant support is available in the area of residence, making it difficult to receive adequate assistance Options for further education and employment in the area of residence are limited	3, 5, 6, 9
	Insufficient information provided by health care professionals and governmental agencies	Insufficient information obtainable from medical professionals The government does not provide sufficient information to families The local government’s consultation system is not well-established, making it difficult to obtain information about support	8, 9
Concerns about the future of the young/adult child	Concerns about the young/adult child’s life after the parents’ death	Anxieties about the future lives of young/adult child Hesitation to discuss issues with the family about what will happen after the parents are deceased Worries about young/adult child’s reluctance to become independent	2, 4, 5, 6, 8, 10
	Concerns about future burden on siblings	Worries about a burden falling on siblings after the parents are deceased Desire that siblings be able to live their own lives Wishing that siblings think seriously about their future	4, 5, 8

**Table 4:** Categories and subcategories of preventive/inhibitory/suppressive factors extracted from the content analysis.

Category	Subcategory	Code	Related Interviews (ID Number in Table 1)
Receiving a diagnosis of the young/adult child’s illness or disability	Family understanding of the causes of the young/adult child’s difficulties	With the diagnosis, family can understand and accept the cause of young/adult child’s difficulties Family feels relieved that young/adult child’s symptoms were correctly diagnosed and treated	3, 8
	Family acceptance of the reasons underlying parenting difficulties related to the young/adult child	Family feels relieved that the diagnosis explains the reasons for the difficulties in parenting young/adult child Family accepts that young/adult child’s clumsiness is “normal,” once the disability has been identified	3, 6, 7
Parental engagement with the young/adult child based on an understanding of their characteristics	Parents adapting their interactions to the young/adult child’s characteristics	Family adapts the family communication to the characteristics of young/adult child Parents understand their young/adult child’s characteristics and increase opportunities to converse with him or her Parents learn about disabilities or characteristics and improve how they interact with their young/adult child	3, 4, 7
	Parenting and support oriented toward the young/adult child’s future independence	Parents always keep in mind their young/adult child’s independence and raise them slightly strictly Parents help their young/adult child develop good habits to enable his or her independence Parents give repeated explanations about sex and hygiene to their young/adult child so they can understand Parents foster self-esteem in young/adult child for his or her future	6, 7, 9
Family maintaining a positive attitude	Positive reframing within the family	Family focuses on the future rather than regretting the past Family focuses on the present rather than worrying about the future Family reframes to utilize support rather than imposing drastic changes on their lives	1, 3, 6, 7, 9
	Family emphasis on the young/adult child’s happiness and well-being	Family approaches young/adult child in ways that will make him or her well-liked by supporters Family wants to support young/adult child so that he or she can grow up happily Family gives optimal priority to the happiness of young/adult child Family wishes for young/adult child’s well-being in his or her future lives	3, 6, 9
	Active family engagement with the surrounding community	Family places value on the interactions with people who have worked with young/adult child Family actively communicates with teachers and support providers Family proactively engages with and builds connections within the surrounding community	3, 9
	Father’s proactive engagement in caregiving and support	Father actively participates in the care of young/adult child Father adjusts his or her work schedules to participate in childcare and support Father and mother repeatedly discuss supporting their young/adult child	1, 3, 4, 9
Adequate support for the family	Availability of caregiving support from relatives	Relatives actively cooperating in the raising of young/adult child Relatives respecting the approach to supporting young/adult child	3, 6
	Proactive engagement by supporters with the family	Supporters actively provide information concerning support Supporters create an environment where family feels comfortable relying on them Supporters engage in activities that foster the strengths of young/adult child Supporters maintain regular contact with the family	1, 3, 6, 9
	Increased functional abilities of the young/adult child resulting from support	Young/adult child learns to think in a logical order through support Young/adult child learns to control his or her emotions through the supporters’ involvement Young/adult child matures to be able to challenge new endeavors by attending support facilities	1, 6
Availability of an environment in which the family can consult others	Parents’ proactive consultation with supporters	Parents actively seek advice from supporters about childcare and support Parents make an effort to consult with supporters even about minor concerns Parents actively participate in childcare and developmental consultations	1, 3, 4, 6, 9, 10
	Parents’ connection with families with similar experiences	Parents participate in parent support groups and gaining information from families with similar experiences Parents interact with families with similar experiences and share their concerns	3, 5, 9

## Data Availability

The raw data used in the study are available from the corresponding author upon request. The data are not publicly available due to privacy restrictions.
